# Psychological resilience training for leaders in extreme times: study protocol of a randomized controlled trial

**DOI:** 10.3389/fpsyg.2025.1514954

**Published:** 2025-09-22

**Authors:** Christina Hersom, Hans Henrik Knoop, Just Bendix Justesen

**Affiliations:** ^1^Department of Educational Theory and Curriculum Studies, Educational Philosophy, and General Education, Danish School of Education, Aarhus University, Emdrup, Denmark; ^2^Department of Educational Theory and Curriculum Studies, Educational Philosophy, and General Education, Danish School of Education, Aarhus University, Aarhus, Denmark; ^3^Department of Sports Science and Clinical Biomechanics, University of Southern Denmark, Odense, Denmark

**Keywords:** psychological resilience, leaders’ resilience, leader resilience, leadership development, building resilience, resilience training, psychosocial resilience training

## Abstract

The fact that organizations face increasing complexity, crises and adverse events requires corporate leaders to respond rapidly and continuously while maintaining their wellbeing and high performance. Psychological resilience is crucial for navigating in extreme times. However, research on building resilience in leader contexts is scarce, particularly regarding how leaders learn to anticipate, cope with, and learn from crises and adversities. This protocol describes a randomized controlled study that examines the dynamics and impact of resilience training focusing both on self-leadership development (psychological resilience) for leading oneself and on leadership development for leading others (psychosocial resilience). Participants include formal leaders and key personnel responsible for leading organizational functions or units. The intervention group will receive resilience training, while the control group will be offered modified training post-intervention. The flexible intervention, grounded in applied positive psychology and cognitive interventions, will be longitudinal, incorporating experiential learning, and involving Human Management Resource (HRM) and educated Human Resource (HR) resilience trainers. Leaders will participate in 20 weekly and collective “resilience-sprints” during extreme times. Primary outcomes will be measured at three time points: before, during, and after the intervention to evaluate effects and explore resilience pathways. Continuous evaluations will identify the relevance of implemented resilience factors, and process evaluations will provide insights into contextual influences and dynamics of resilience building. The study integrates individual and organizational factors into a psychosocial resilience intervention designed as a comprehensive leader training program. The study protocol directs a study that aims to enhance empirical understanding of building leader resilience in extreme times of crisis and adversities, to benefit research in Management, HRM, and resilience fields. Ultimately, the study aim to help leaders face, cope, and adapt effectively by learning from experiences with the complexities of adverse and pressured organizational contexts.

## 1 Introduction

The concept of resilience is necessary for understanding how organizations and leaders respond successfully to crises, unexpected events, and significant adversities. Extreme times, marked by prolonged crises and persistent stressful challenges, underscore its importance. For instance, the COVID-19 pandemic severely disrupted organizational processes and activities, forcing leaders to deliver positive results during prolonged and extraordinary challenges (Țiclău et al., 2021). Since then, crises and unexpected events have increasingly exerted a significant and lasting impact on organizations, necessitating rapid adaptation to ever-changing environments. Recent perspectives on resilience emphasize its multifactorial nature and contextual dependence, which is crucial for understanding how leaders can effectively manage adversity in complex organizational environments (Cooper et al., 2013; Förster and Duchek, 2017; Förster et al., 2022c). Unlike traditional views that consider resilience merely as an individual trait or the ability to “bounce back,” current management research advocates for a broader appreciation of distinct organizational responses to adversity that goes beyond stability as it incorporates thriving and growth from adversity (Munoz et al., 2022). Combined perspectives includes resilience as a dynamic process and outcome involving both individual, situational, and behavioral resilience factors (Förster and Duchek, 2017) unfolded during crises where leaders must anticipate, cope, and adapt (learn) from different demands and challenges to reach high levels of resilience (Förster and Duchek, 2022; Lombardi et al., 2021). Organizations can either enable or hinder resilience through their structural and cultural frameworks, with leadership practices playing a crucial role in shaping these environments. In this perspective, leader resilience is not only positively related to follower performance (Walumbwa et al., 2010), it also creates a culture of well-being and work productivity, significantly enabling or moderating resilience for team members and the organization (Förster and Duchek, 2017). During crises, leader resilience positively affects organizational resilience (Prayag et al., 2024; Țiclău et al., 2021) and employee resilience (Zhang et al., 2024; Singh et al., 2023) serving as social, emotional, and cognitive resources for organizational resilience (Teo et al., 2017). Leaders thus have a great influence in organizations, but importantly, they should also invest in their own resilience (Mann et al., 2021). Psychological leader resilience affects not only their own performance but also sustainability, so those who develop leaders, should create safe environments to help leaders thrive as individuals and as organizational leaders with resilience, which is directly related to the stress of the leader’s job (Ledesma, 2014). Despite this importance of resilience in leader contexts, the research field lacks sufficient knowledge about what makes leaders resilient (Förster and Duchek, 2017; Scheuch et al., 2021; Ledesma, 2014). Some insightful studies have explored leader-enhancing factors in various organizational contexts (Förster and Duchek, 2017; Förster et al., 2022b; Ukhova, 2020; Förster and Duchek, 2022). However, when it comes to learning and building the necessary capacity, meta-reviews uncover a scarcity of empirical evidence on building psychological resilience in leader contexts (Robertson et al., 2015; Vanhove et al., 2016). This has prompted urgent calls for research in managerial resilience training settings and highlighted its necessity due to current adverse circumstances (Scheuch et al., 2021). This training may draw on experience-based learning methods (Förster and Duchek, 2022) and strategic Human Resource Management (HRM) must develop resilience knowledge, skills, and abilities at the individual level that, when aggregated at the organizational level, enable collective intelligence and behavioral preparedness (Lengnick-Hall et al., 2011; Liu et al., 2021). Building on such knowledge, this study concentrates on developing and implementing supportive resilience resources that empower leaders to enhance their adaptive responses, linking psychological and psychosocial resilience with leadership through targeted training in extreme times. The leader-centered resilience intervention embraces what has been stated as one of the paradoxes of human psychology: vulnerability to pressure and adversity is needed for developing the resilience necessary to confront these challenges and achieve high performance (Fletcher and Sarkar, 2016). Here, vulnerability and resilience coexist as leaders must initially yield to adversity derived from their daily work to gain from the psychological and behavioral changes that only organizational context-induced pressure can produce. This intervention approach builds on the concept of “antifragility” (Taleb, 2013) operationalized as a core training principle, suggesting that systems and actors can thrive and grow stronger specifically when they are exposed to stressors. Antifragile systems, such as a leader’s cognitive, emotional, social, and behavioral processes, require exposure to adversity to learn, adapt, and grow, thereby enhancing their ability to successfully navigate future uncertainty. Conversely, avoiding such events can lead to rigidity, weaknesses, and inefficiency, lacking the necessary challenges to prompt a dynamic response (Lukianoff and Haidt, 2018). Today, leaders are constantly exposed to adversity and unexpected events that create significant tensions, requiring them to balance competing demands while capitalizing on opportunities (Förster et al., 2022c; Wu et al., 2021). The intervention in this study trains leaders to navigate various types of adversity by adapting both mindsets and behavior, developing resilience for dealing with tensions and paradoxes (Lewis and Smith, 2023). The intervention incorporates exposure to leaders’ natural environments while facilitating structured, collective learning, enabling them to navigate relevant processes of uncertainty and stressful situations. The study then operationalizes the paradox of human psychology as “the paradox of leader resilience,” highlighting that adversity-triggered vulnerability can create opportunities for leaders, such as exerting greater influence over their tasks and enhancing their psychological work environment (Johansen, 2021). Even a crisis can become a turning point for positive outcomes when managed effectively (Förster et al., 2022c; James et al., 2011). Stressful situations can serve as catalysts for growth and optimal functioning when individuals proactively seek adversity and cultivate resilience skills, ultimately enhancing well-being and mental health (Seligman, 2011). By becoming conscious, awake, and mindful during coping moments, leaders can build up a repertoire of proactive strategies for future adversities (Elkington and Breen, 2015). Consequently, the collective leader training intervention in this study anticipates that leaders will be able to transform moderate exposure to stressful situations by cultivating skills and shared resilience strategies for effective adaptation. This cultivation of resilience for leading oneself and others is expected to impact resilience, mental health, well-being, and psychosocial functioning as demonstrated in previous resilience training studies for employees in organizations (Robertson et al., 2015; Scheuch et al., 2021; Chitra and Karunanidhi, 2021). Both resilience and psychological well-being are important outcomes, as suggested by resilience researchers in leader contexts (Förster et al., 2022b). As an empirical study, the research study goes beyond merely evaluating outcomes by establishing a comprehensive organizational training environment. Here, leaders can collectively build resources over time by confronting adversities across diverse leadership contexts. Processes reflecting psychological resilience capacity depend on individual, social, and environmental resources (Britt et al., 2016), which in this training study are provided and encouraged over an extended period. Viewing resilience as a reciprocal responsibility means organizations must provide a context for promoting resilience, while leaders need to utilize available resources to advance and sustain resilience capacity (Kuntz et al., 2016). By providing this collective resilience-promoting environment, the study aims to build resilience capacity across multiple managerial levels, responding to calls for resilience research in leadership contexts (Ukhova, 2020; Scheuch et al., 2021). Addressing this research gap, the study contributes to bridging psychological and psychosocial resilience with resilience factor literature in leader contexts, highlighting a variety of common resilience factors. Furthermore, the study addresses the pressing need to understand how resilience can be interconnected across different levels (Raetze et al., 2021), by contributing an operationalized multilevel resilience approach derived from a Human Resources (HR)-driven resilience training program designed for communities of leaders.

Consequently, this study hypothesizes the following:

1. Leaders who receive the resilience training intervention will report reduced levels of perceived stress and either sustained (resilient) or improved (growth) levels of psychological resilience, work-related resilience, mental health, and well-being outcomes, compared to the control group.
2. Leader resilience training will have a measurable impact on learning and development outcomes. Additionally, by examining impact-dynamics, implementation strategies, and various context-dependent influences, the study will reveal how individual and situational/environmental resources contribute to leader resilience processes and behavioral leadership outcomes during extreme times.

## 2 Method

This study protocol outlines the foundational resilience model, theoretical underpinnings, resilience framework, implementation procedure, and change model for developing resilience in leadership contexts. It also provides a detailed description of the research design and measurement methods, followed by a discussion of the study’s implications.

**Figure 1:**
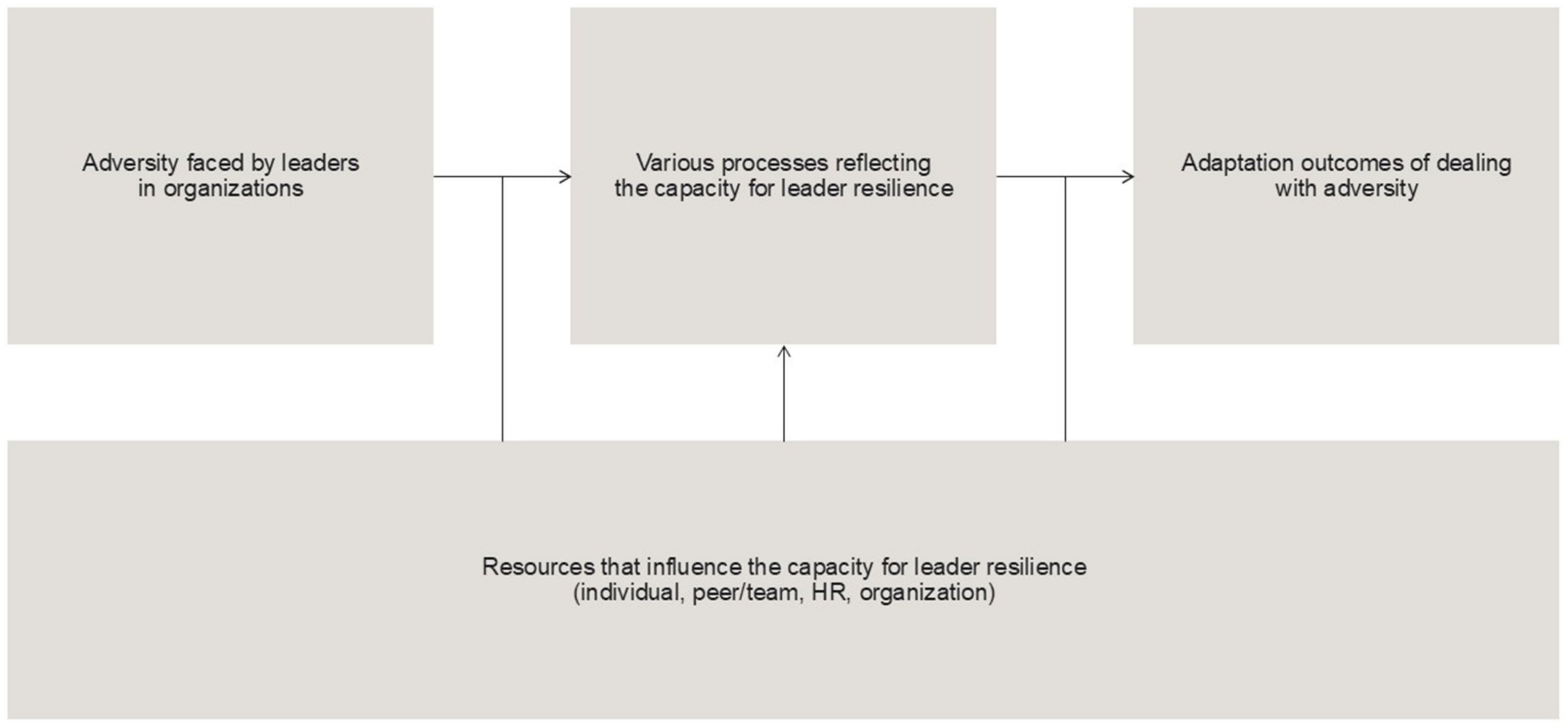
Main components of the foundational leader resilience model, adapted with permission from Britt et al. (2016).

Methodically, there is a growing need for well-designed controlled studies that investigate resilience building in leader contexts (Scheuch et al., 2021; Ledesma, 2014). This study responds to these calls by implementing a multi-wave randomized controlled trial (RCT) to examine the effects of resilience interventions in leader contexts during extreme conditions in organizations. We also explore how implemented resilience factors and training mechanisms affect learning and development processes, as well as the influence on contextual factors. Understanding the effects of resilience training in leader pressured contexts and the underlying mechanisms is crucial, given the existing knowledge gap on building leader resilience and the complex high-pressure conditions leaders must face today. The resilience training aims to enhance skills and strategies for effective behavior in adverse and challenging leader contexts (Förster and Duchek, 2017; Förster et al., 2022c). The study is informed by an integrative resilience model based on psychological resilience interventions identified across multiple organizations. This model enhances conceptual clarity by incorporating significant adversity and distinguishing between the capacity for resilience processes and the demonstration of resilience in terms of health and performance outcomes (Britt et al., 2016). This approach emphasizes capacities that can be developed and the outcomes of these resilience processes, influenced by internal and external resources, and specifically supported in this study from individuals, colleagues/teams, HR-trainers, and organizational management – thereby contextualizing leader resilience development. This integrated process and outcome approach aligns with current resilience research in leader contexts (Förster et al., 2022b; Förster et al., 2022c) by directly including individual, situational/organizational, and behavioral factors, when individual leaders train the behavioral lead of themselves and others in various challenging situations in the organizational context. The foundational psychological resilience model tailored for the leader study is illustrated in Figure 1, showing how leaders need to face adversity and engage in resilience processes leading to various outcomes. These processes are influenced by contextual resources within the organizational training and development environment.

The resilience study aims to equip leaders with various resilience opportunities in two large organizations (private and public) headquartered in Denmark. The operational objective of the study involves designing, implementing, and conducting the contextualized and comprehensive leader resilience training intervention that adopts a multidisciplinary, multimodal, and multilevel supportive approach. The study contributes with empirical knowledge on collective resilience training in leader contexts for leaders leading themselves and others and pinpoints implications for future empirical resilience interventions.

### 2.1 An interdisciplinary framework for the leader resilience intervention

Addressing the paradox of psychological leader resilience, it is essential in management research to know the overlap between resilience and associated but distinctly different concepts and responses to adversity (Munoz et al., 2022). In this view, resilience can be contrasted with “robustness,” which represents insensitivity to uncertainty and resistance to adversity, providing short-term relief but potentially rendering leaders more vulnerable and inflexible over time, making it harder for them to succeed in their demanding roles. Additionally, resilience differs from the concept of antifragility, as resilience may involve performance degradation followed by “bouncing back” from adversity, whereas antifragility refers to thriving and growing stronger in response to stressors (Munoz et al., 2022). However, within research focusing on human resources in organizations, resilience is understood as a combination of bouncing back from setbacks, effectively facing tough demands and difficult situations, and growing stronger in the process (Liu et al., 2019). In this study, we also utilize an integrated interdisciplinary approach for the resilience intervention to address different circumstances and mechanisms in response to occurring situations.

To build leader resilience, the study adopts a central approach from positive psychology, rooted in humanistic psychology (Maslow, 1954; Rogers, 1951) and Aristotelian philosophy of eudaimonia, emphasizing well-being and meaning in life (Seligman and Csikszentmihalyi, 2000). Positive psychology shifts focus from treating psychopathology to promoting character strengths, positive experiences, and organizational contexts to optimize human potential (Seligman and Csikszentmihalyi, 2000; Snyder et al., 2002). This shift is operationalized by moving beyond risk factors and treating dysfunctional or stressed leaders toward promoting and developing available resources and resilience capacity for leaders and organizations to sustain thriving and optimal functioning under challenging circumstances (Cooper et al., 2013), as we believe challenges, adversities, and crises will probably not disappear. Consequently, this realistic approach is applied in the study as “psychological leader resilience,” advancing scholarly research by focusing on self-leadership development (psychological resilience) for leading oneself and on leadership development for leading others (psychosocial resilience), as this is highly intertwined. Positive psychology will be operationalized through various collective interventions tailored for leaders, drawing from previous research on emotional regulation, optimism, and authenticity for human strengths, wellbeing, and resilience purposes (Reivich et al., 2011; Niemiec, 2018; Fredrickson, 2004). An interdisciplinary approach is beneficial in the study of resilience in organizations, including the trainability and effectiveness of incorporating Cognitive Behavioral Therapy (CBT) techniques in building resilience (Reivich and Shatté, 2002; Padesky and Mooney, 2012). These techniques for cognitive flexibility during stressful situations have been successfully applied in multimodal resilience-building programs for diverse populations (Millear et al., 2008; Chitra and Karunanidhi, 2021; Liossis et al., 2009; Coleman et al., 2020; Burton et al., 2010). This part of the intervention includes brief insights on thinking patterns and mental strategies, tailored to promote *mental awareness* and cultivate *flexible change skills* necessary for resilience in organizational settings (Reivich and Shatté, 2002). Leaders need to be mentally ready completely aware of the crucial role they have in teams and organizations, particular in times of crises (Förster et al., 2022a). We therefore combine positive psychological interventions with validated CBT techniques CBT (Beck, 1989; Ellis, 1994) to strengthen the theoretical and interdisciplinary framework for leaders to draw on a broad spectrum of resilience strategies to navigate the complex, unexpected, and diverse situations they need to face. The program will offer various options for testing resilience strategies for thinking, feeling, and behaving as leaders in their pressured leadership roles.

Drawing from the above theoretical foundation and associated resilience factors, the resilience program incorporates three core components: (1) a character strength-based component, (2) an emotional/interpersonal component, (3) a cognitive component. This multidisciplinary program builds on psychological/psychosocial factors supported by strong evidence as appropriate determinants of resilience across different populations (Helmreich et al., 2017), and many of these factors are similarly identified in leader contexts (Förster and Duchek, 2017; Ukhova, 2020). For instance, being optimistic, self-confident, and flexible, demonstrating communication and social skills, and behaving reflectively and analytically are important in both leadership and organizational contexts. Such qualities are embedded in the program, where leaders are continuously given opportunities to exchange with kindred spirits. It leverages previous resilience training programs that have implemented such resilience factors in organizational settings (Las Hayas et al., 2019; Chitra and Karunanidhi, 2021; Millear et al., 2008; Burton et al., 2010; Reivich et al., 2011; Fletcher and Sarkar, 2016) and in resilience training studies showing mental well-being, psychological health, and performance outcomes in non-clinical populations (Robertson et al., 2015; Sarkar and Fletcher, 2017; Vanhove et al., 2016). The three core components are operationalized in this study through a framework of (1) My strengths, (2) My team, and (3) My inner. To serve as a replicable reference, the program content is presented in Appendix 1. This contextualized resilience program aims to systematically equip leaders with resilience skills and strategies tailored to the unique demands of their “double-leading role” of leading both themselves and others in pressured organizational contexts.

### 2.2 Implementation of the leader resilience training program

The comprehensive framework for leader training includes a curriculum with 18 subcategories derived from the three multidisciplinary core components, covering a wide range of adaptation opportunities. The program is divided into two parts: Part I includes 18 basic training sessions, and Part II comprises 18 advanced sessions, totaling 36 pre-designed training sessions. This division ensures a progressive approach and macro program flexibility, as demonstrated in previous studies (Ungar and Jefferies, 2021; Las Hayas et al., 2019). To tailor the curriculum to the specific needs of participant organizations, a co-creation process will be employed. This involves collaboration with participant organizations to align the training program with their local visions and resilience needs. The selection of the final curriculum will be based on local aggregated baseline assessment data and will follow specific workshop protocols. The contextualized curricula will consist of 20 weekly one-hour resilience training sessions (embedded in weekly resilience-sprints), with 10 sessions in each part (Part I and Part II). Recognizing that resilience development requires time and effort, the program is designed as a prolonged training intervention, emphasizing reflection and experiential engagement in fostering resilience (Masten, 2011; Elkington and Breen, 2015; Cooper et al., 2013).

The program will be implemented as a multi-level supportive initiative, incorporating resources from individuals, leader colleagues, HR, and management in participant organizations. We believe the integration of trained internal HR facilitators and co-creation of the curriculum increases ecological validity and implementation potential. The responsibility for resilience should be shared among stakeholders (Ungar and Jefferies, 2021), and our community-based approach integrates both individual and organizational resilience promoting factors identified within leader contexts (Förster and Duchek, 2017; Ukhova, 2020). For instance, the internal HR-resilience trainers will also undergo intensive resilience training themselves following specific educational protocols. This ensures that trainers are well-prepared and understand the program from a participant’s perspective before implementing the resilience training within their organization. The goal is to ensure an ethical and sustainable supportive learning environment for leaders. During implementation, we will use a unique method that we in this study term “intelligent psychological resilience training” to address the paradox of psychological leader resilience. This approach draws inspiration from community-based programs promoting individual physical health in organizational contexts (Sjøgaard et al., 2014; Dalager et al., 2016). This method underscores the importance of incorporating the two core components of resilience: adversity exposure and positive adaptation, tailored specifically to the individual. These components play a crucial role in building resilience capacity in training programs where challenges and adversity are needed (Vanhove et al., 2016). Exposure to moderate adversity is associated with resilience when facing controlled stressors and creating coping-related benefits (Seery et al., 2013). Therefore, participants will receive adversity-infused training in navigating and adapting to challenges based on their own selected and listed adverse experiences from the leader context to ensure ecological validity. This training will align specific exposure levels within the program’s scope and consider how participants perceive paradoxes and tensions differently (Lewis and Smith, 2023). Participants will be guided to set individual weekly learning attentions and goals to engage in self-directed learning processes between sessions (Knowles, 1980). They will be encouraged to apply resilience skills and behaviors in their leadership context and then discuss and evaluate the outcomes of various resilience strategies with HR-trainers and colleagues from the training community in subsequent sessions. This peer exchange is critical for leader resilience in organizational contexts (Förster and Duchek, 2017). Through an experiential-based approach (Kolb, 2014), the study thus aims to provide continuous and iterative learning opportunities for leaders, enabling them to apply resilience in interaction with natural environments when leading themselves and others through extreme times. Discussions will focus on cognitive, affective, and behavioral processes that constitute organizational leader resilience resources, integrating individual and organizational levels (Raetze et al., 2021). The longitudinal approach allows us to study the effects of resilience training over time, as recommended by previous reviewers (Robertson et al., 2015; Vanhove et al., 2016).

**Figure 2:**
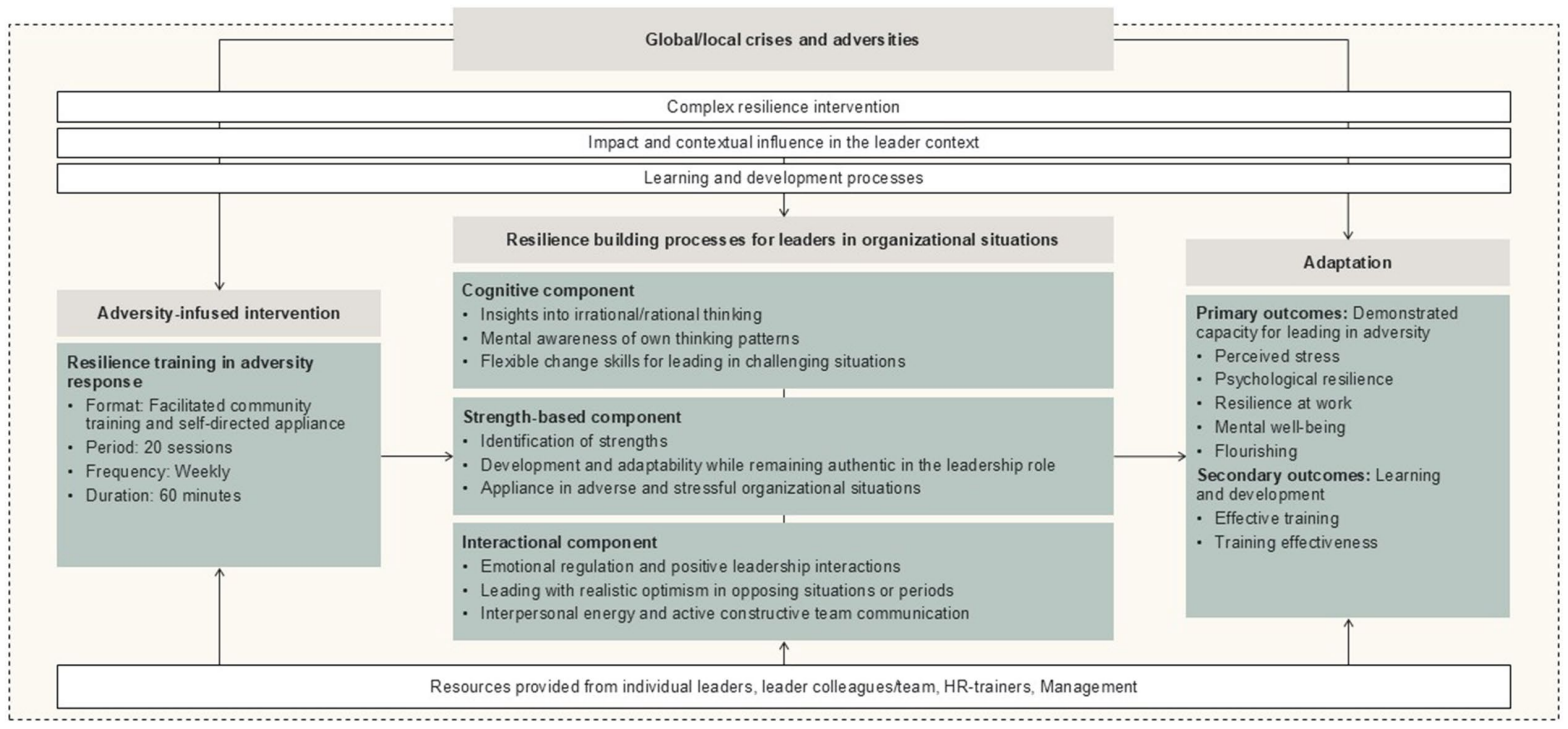
Overall framework for the leader resilience intervention.

Figure 2 illustrates the framework for building leader resilience, which is based on the foundational resilience model, the multi-disciplinary theoretical framework, and multimodal training. This figure shows the complete logical model and aligns the data sources used in this study.

### 2.3 Research design

Consistent with recommendations from the latest comprehensive meta-review of resilience training in organizational contexts (Scheuch et al., 2021), this study is a randomized controlled trial (RCT) to examine the impact of behavioral resilience training in leader contexts. The majority of participants are recruited from the private organization, due to massive resource planning in the public organization. Following prior organizational resilience studies (Vanhove et al., 2016), we will assess primary outcomes to demonstrate resilience capacity (Britt et al., 2016) and evaluate dynamic resilience pathways over time (Masten and Reed, 2002), in this study through three measurements. Resilience in response to stressors of varying intensity is expected to show different outcome trajectories, allowing for more accurately observation and assessment in the context in which it occurs (Pangallo et al., 2015). To achieve this, participants will complete questionnaires at multiple time points: before (T0), during (T1), and after the intervention (T2). This longitudinal analysis will provide insights into resilience dynamics in leader settings and evaluate outcomes after the extended intervention, as suggested by several resilience researchers (Vanhove et al., 2016; Robertson et al., 2015; Ukhova, 2020). For secondary outcomes of interest, using a mixed-method design, we will assess training effects via the Kirkpatrick model (Kirkpatrick and Kirkpatrick, 2016), which also allows indirect analysis of the implemented resilience factors. Additionally, we will conduct a process evaluation of contextual conditions during and after the intervention (T1, T2) and explore participant experiences using various process evaluation frameworks proposed by intervention researchers (Moore et al., 2015; Randall et al., 2007; Braun and Clarke, 2006). The logical model for the framework variables and data sources used in the learning and development evaluation part of the study is mapped in Appendix 2. The overall aim is to evaluate the effects, processes and underlying mechanisms of the intervention, providing insights into what, why, and how of training psychological resilience in leader contexts. The total research design is outlined in Table 1 according to SPIRIT 2013 recommendations for standard protocols (Chan et al., 2013) showing different research and implementation activities.

**Table 1:** The schedule of enrolment, interventions, and assessments.

Study period							
	Enrolment	Allocation	Baseline	Intervention (Part I)	During	Intervention (Part II)	Follow-up (< 3 months)
TIMEPOINT		0	T0		T1		T2
ENROLMENT							
Eligibility screen							
Informed consent							
Allocation							
INTERVENTIONS							
Resilience training intervention (20 sprints)							
No intervention (control group)							
ASSESSMENTS							
Socio/ demographic variables: age, gender, education, job function, seniority, occupation, responsibility, functional area							
Primary outcome variables: Perceived stress, CD-RISC 10, RAW, WEMWBS, Flourishing							
Secondary outcome variables: training evaluation (Kirkpatrick x 20)							
Secondary outcome variables: process evaluation (self-constructed scales)							

### 2.4 Participants and recruitment process

Participants for this RCT study are recruited from public and private organizations within a management consultancy network in Denmark, including national and international leaders. The recruitment process involves contacting HR Management via email or phone and introducing the program to interested organizations. Interested HR managers receive materials to engage internal unit managers, who is invited to participate if they express interest. To ensure unbiased selection, all leaders from enrolled units are invited to voluntarily sign up for the local resilience training program.

Inclusion criteria:

- Age 18 or above
- Formal leadership role or indirect leadership responsibility
- Willingness to participate in an RCT study and complete anonymous questionnaires
- Commitment to the resilience program
- Attendance of weekly 1-h sessions during the intervention period
- Engagement in resilience training within a peer community

Exclusion criteria:

- Late sign-up after the recruitment period
- Being on sick leave during recruitment and introduction
- Lack of commitment to the scheduled training intervention
- Withdrawal of consent to participate

### 2.5 Randomization of participants

Following guidelines from a previous meta-review on resilience training in organizational contexts (Robertson et al., 2015), individual participants from organizational units will be randomly assigned to either the experimental or control group using computer-generated simple randomization. To mitigate contamination risks, the intervention group will from the beginning be advised not to share intervention content and training materials with the control group. HR-business partners will monitor the participating units, where leaders also work across the organization in many diverse team compositions, which we expect can reduce direct interaction, intervention conversations, and spill-over effects. Both groups will continue with their regular job responsibilities; however, the experimental group will also participate in weekly training sessions and additional self-directed training between sessions. The control group will only complete questionnaire measurements before, during, and after the intervention. After the final measurement, control group participants will be offered a condensed period of resilience training. This behavioral intervention study will be reported using an extension of the CONSORT Statement for social and psychological interventions (Montgomery et al., 2018).

### 2.6 Materials

#### 2.6.1 Qualifying the measurements tools

To ensure contextual relevance, all outcome measurements, curricula, materials, and activities will be aligned with corporate language requirements, utilizing original and translated validated questionnaires for both Danish and English-speaking organizations. One necessary questionnaire will be translated into Danish following the forward-and back-translation guidelines (Beaton et al., 2007). Further, we will employ the Rasch methodology (Rasch, 1960) to assess the psychometric properties of the questionnaires. Recognized in Business and Management Studies for handling subjective, ordinal, and non-observable variables (García-Pérez and Yanes-Estévez, 2022), the Rasch model allows for constructing a unidimensional scale based on participants’ evaluations and testing the fit of the sample data to this model (Stemler and Naples, 2021). Using the software package RUMM2030 (Andrich et al., 2009), we will specifically use the Polytomous Rasch model that examines the likelihood of individual leaders providing endorsement responses to each test item, considering unequal difficulty levels of endorsement across test items (Boone et al., 2014).

#### 2.6.2 Primary outcomes

In this study, we adopt a comprehensive approach to assess the capacity and demonstration of resilience by employing two distinct measurement strategies. We directly measure resilience outcomes using specific resilience scales and, further we assess resilience-related outcomes, signaled by wellbeing and performance indicators (Vanhove et al., 2016). In total, this assessment design aligns with our foundational resilience model focusing on outcomes relevant to resilience in organizational settings (Britt et al., 2016; Kuntz et al., 2016).

##### 2.6.2.1 Perceived stress

To assess participants´ perceived stress levels and their coping abilities, resources, and sense of control in the period, we will use the 10-item Perceived Stress Scale (PSS-10) (Cohen et al., 1983). This scale includes both negatively phrased items (e.g., “In the last month, how often have you felt nervous and stressed?”) and positively phrased items (e.g., “In the last month, how often have you felt that you were on top of things?”). Participants will rate their agreement with each statement on a 5-point Likert scale, ranging from 0 (never) to 4 (very often). The total score, ranging from 0 to 40, will be calculated by reversing the ratings for the positively phrased items. Higher scores indicate a higher level of perceived stress. The Danish version of the scale has been previously validated (Olsen et al., 2004) and used in research by the National Institute of Public Health in Denmark (Nielsen and Randall, 2013).

##### 2.6.2.2 Psychological resilience

We will use the CD-RISC 10 scale, a shortened version of the original 25-item CD-RISC (Connor and Davidson, 2003; Campbell-Sills and Stein, 2007), to assess resilience as a personality trait for effectively coping and thriving in the face of adversity. The Danish version of the scale was obtained from a previous translation (Lauridsen et al., 2017) for research purposes. This scale measures several aspects of resilience, including flexibility, self-efficacy, emotional regulation, optimism, and cognitive focus under stress. Participants rate each item on a 5-point Likert scale, from 0 (not true at all) to 4 (true nearly all the time), with total scores ranging from 0 to 40. A higher total score indicates a higher level of resilience in individuals.

##### 2.6.2.3 Resilience at work

To measure resilience at work, we will use the RAW scale (Winwood et al., 2013), which assesses resilience in the context of emotional distress. Resilience is viewed as a dynamic resource that can be developed at work through targeted skill training, emphasizing that resilience behaviors and strategies are not solely determined by genetic and personality factors. The RAW scale includes 20 items covering seven aspects: energy, self-care, adaptability, purpose, network, authenticity, and support. Participants rate each item on a 7-point Likert scale, from 1 (strongly disagree) to 7 (strongly agree), with higher scores indicating higher resilience capacity at work. The RAW scale will be translated into Danish with permission from the authors and following recommended guidelines (Beaton et al., 2007; Sousa and Rojjanasrirat, 2011). Further, it will be validated through Rasch analyses to ensure its psychometric properties and appropriateness for the resilience study.

##### 2.6.2.4 Mental well-being

We will assess mental well-being using the Warwick-Edinburgh Mental Well-Being Scale (WEMWBS) (Tennant et al., 2007). This scale includes 14 positively worded items covering hedonic (feeling) and eudemonic (functioning) aspects such as positive affect, satisfying interpersonal relationships, and positive functioning. Participants rate each item on a 5-point Likert scale, from 1 (none of the time) to 5 (all the time), with total scores ranging from 14 to 70. Higher scores indicate higher mental well-being. The Danish version has been used in the European Social Survey (ESS) and validated by the National Institute of Public health in Denmark (Nielsen and Randall, 2013).

##### 2.6.2.5 Flourishing

To measure positive mental health and well-being, we will use the 10-item Flourishing scale (Huppert and So, 2013), which assesses hedonic and eudemonic dimensions including competence, emotional stability, engagement, meaning, optimism, positive emotions, positive relationships, resilience, self-esteem, and vitality. Most items are rated on a 5-point Likert scale, from 1 (strongly disagree) to 5 (strongly agree). Exceptions are positive emotional stability and vitality rated on a 4-point Likert scale, from 1 (none or almost none of the time) to 4 (all or almost all of the time) and positive emotions, rated from 0 (extremely unhappy) to 10 (extremely happy). Higher scores indicate higher levels of flourishing. The Danish version has been used in the European Social Survey (ESS) and validated by the National Institute of Public health in Denmark (Nielsen and Randall, 2013).

#### 2.6.3 Secondary outcomes measurements

During the training period, the intervention group will engage in continuous evaluation of the resilience training. The aim is to better understand the processes and mechanisms of change when training resilience in extreme times with leaders.

##### 2.6.3.1 Evaluation of implemented resilience factors and training effectiveness

The program incorporates subcategories derived from the three main resilience components, allowing us to explore their relevance as training mechanisms. For this, we use the Kirkpatrick scale (Kirkpatrick and Kirkpatrick, 2016), a widely recognized evaluation model applicable in various training contexts (Alsalamah and Callinan, 2022) and for leadership development (Lacerenza et al., 2017). This scale includes four levels: reaction, learning, behavior, and results indicating effective training (Level 1–2) and training effectiveness (Level 3–4) (Kirkpatrick and Kirkpatrick, 2022). We operationalize this flexible model into a simple four-item measurement supplemented by an open-ended item for comments or qualitative responses. At the start of each training session, participants will reflect upon their adverse experiences and coping behaviors since the last session. They will then share and discuss resilience strategies with peers and trainers to learn from it. Finally, they will evaluate their weekly training, forming a cyclic learning and development process termed the “leader resilience-sprint.” The objectives of this evaluation part, include identifying the most relevant resilience-promoting factors from a training content perspective, understanding the training and learning mechanisms during intervention, and providing self-monitored progress feedback to potential enhance motivation, goal pursuit, and reduce dropout rates (Locke and Latham, 2019; de Jong et al., 2021).

##### 2.6.3.2 Process evaluation of influencing factors on leader resilience processes

Aligned with previous recommendations (Robertson et al., 2015), we explore factors influencing change during resilience training viewing resilience as dynamic and shaped by contextual and person-environment interactions (Förster and Duchek, 2017; Robertson et al., 2015). To identify the individual and organizational mechanisms that may hinder or facilitate intervention change (Nielsen and Randall, 2013), we will conduct a process evaluation that adapts to the specific intervention context (Arends et al., 2014). Data is collected from questionnaires at two timepoints (T1 and T2), as process elements may evolve over time (Nielsen and Randall, 2013). The questionnaires, discussed within the research team, are outlined by micro-processes (intervention implementation), macro-processes (design, delivery, maintenance), and intervention context (organizational and external conditions) following a guiding framework for analyzing process data (Randall et al., 2007). Process evaluations can complement effect evaluations in a mixed method design (Nielsen et al., 2010), helping to explore factors impacting the intervention outcomes (Nielsen and Randall, 2013). Furthermore, at T2, we will assess the intervention’s usability through open-ended questions, gathering examples on how leaders applied their learnings and resilience strategies impacting leader resilience practices. We will also assess participant attitudes to evaluate feasibility, acceptability, strengths, and areas for improvement of resilience training in leader contexts. A thematic analysis of participant experiences (Braun and Clarke, 2006) integrated into a framework for analyzing complex interventions, focusing on context, implementation, and mechanisms of impact (Moore et al., 2015) will be used to interpret this data, conceptualizing, and informing future resilience training initiatives. Together, these different process evaluation approaches aim to elucidate the *why* and *how* leader resilience training works, complementing the *what* from the statistical outcome evaluation.

### 2.7 Data management and statistical analysis of results

**Figure 3:**
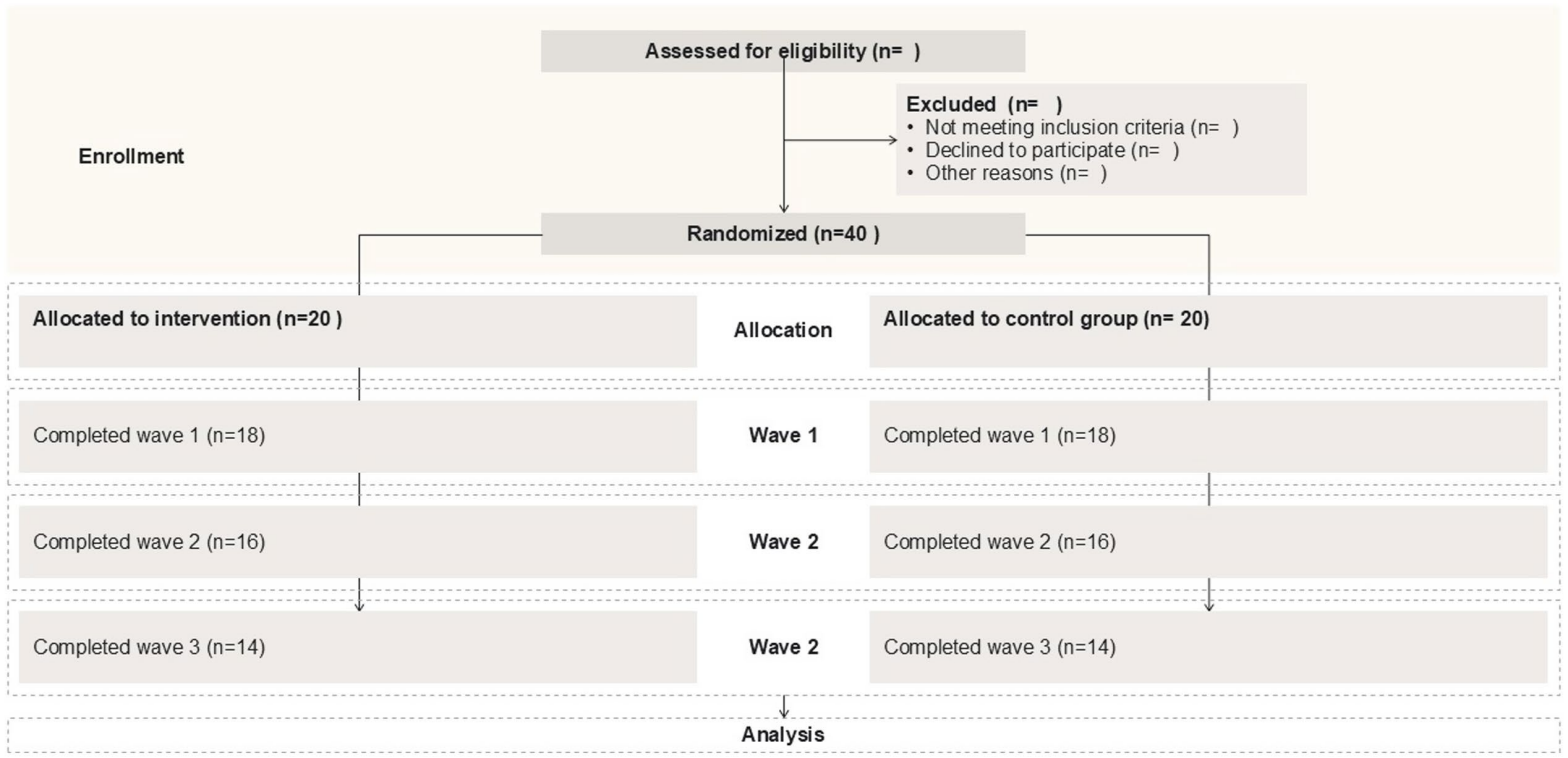
CONSORT flow diagram for attrition.

*A priori* power calculations were conducted using G*Power 3.1 (Faul et al., 2007) to determine the required sample size for a repeated measure and ANOVA assessing the interaction between time (three waves) and group (intervention versus control). The analysis for repeated measures, within-between interaction, was based on the following parameters: a medium Cohen’s effect size (*f* = 0.25), a significance (Alpha) level of 0.05, desired power of 0.08, and correlation among repeated measures of 0.5 for two groups and three measurements. The power analysis indicated that a total sample size of 28 participants (14 per group) was deemed necessary to achieve 80% power to detect a group x time interaction in this multi-wave organizational trial. Potential attrition across waves is accounted for as indicated in the CONSORT 2010 Flow Diagram in Figure 3, illustrating sample size planning for 30% attrition, assuming attrition occurs evenly from recruitment and across the waves with a rate of approximately 10% throughout the study. However, the aim is to increase the final target sample size to approximately 100 participants to enhance the validity of the study, due to pressures from extreme times in the organizations.

Propensity scores, calculated via logistic regression, will ensure balanced covariate distribution between intervention and control groups, enhancing the validity and reliability of the randomized controlled study (Rosenbaum and Rubin, 1983). Participants will complete secure online questionnaires via Survey X-act, collecting sociodemographic data (age, gender, education) job-related factors (function, seniority, occupation, responsibility, functional area), and outcome data. Aggregated local baseline assessments (T0) will guide need analysis and curriculum adjustments, as recommended by reviewers of leadership training, to optimize learning and transfer (Lacerenza et al., 2017). Internal HR-trainers will be blinded to subsequent intervention data (T1, T2) to minimize training biases. Growth modeling procedures in leadership training have proven effective in evaluating leader changes during interventions (Day et al., 2021). SAS PROC MIXED (SAS Institute Inc, 2022) will be used to fit the Growth Model. An unconditional model will predict longitudinal data, followed by conditional linear growth models examining interaction effects (*p*-values) of person-level covariates on short-term (T0, T1) and long-term (T0, T2) trajectories for all primary outcome variables. Rasch analysis will calculate Cronbach’s Alpha (reliability), item fit, eigenvalues (dimensionality), and differential item function (DIF). Process evaluation data will be analyzed using the package “Markov chain” (Spedicato, 2017) for discrete time Markov chains, examining the progression of Kirkpatrick levels and relevant resilience factors implemented over time. Descriptive analysis will be performed using R (R Core Team, 2024) to show influencing factors on the leader resilience training and resilience processes. Participant data from Survey X-act will be managed by a designated research team member using unique participant codes and analyzed by an external statistician to reduce research biases. Data retention is set for 5 years post-study.

## 3 Discussion

This study addresses a significant gap in management research by focusing on resilience in leader contexts (Förster and Duchek, 2017). Given the increasing prevalence of leader stress and challenging circumstances, this study will allow for the examination of this important yet understudied population by leveraging both individual and environmental resilience resources, acknowledging the interplay between these factors (Förster and Duchek, 2017; Ukhova, 2020). The study recognizes that resilience building in organizations is a collective responsibility (Ungar and Jefferies, 2021; Kuntz et al., 2016) important for the strategic HRM agenda and initiative. Management and HR are therefore responsible for structuring and allocating educated resources for weekly resilience sprints and internal resilience trainer teams that are actively engaging in the intervention process. The research team serves as an external resource, providing guidance on content, delivery, and emerging issues. This collective and involved approach addresses a need for internal resilience training capacity, especially given the novel focus on resilience for HR and leaders accustomed to more traditional leadership development programs (Day et al., 2021). Even though the intervention’s focus on interactional psychological resilience processes may limit its direct attention to the broader environmental conditions, the individual level is recommended as a point of departure for organizational resilience (Lengnick-Hall et al., 2011). Ultimately, the study will support and equip leaders with strategies to apply resilience in their daily interactions and processes to maintain or develop wellbeing and performance in extreme times.

The paradox of psychological leader resilience is central in organizations, as leaders face both risks and opportunities. The concept of resilience, with its core component of adversity, sheds light on the stressful conditions faced by leaders, further contributing to the de-tabooing of leader stress in organizational environments (Skakon et al., 2011). The study will improve empirical understanding of the impact of structured and collective resilience building in extreme times from a multilevel HR integration that increases translational value. During the extended period, leaders are encouraged to face moderate challenges from their natural environments with consciousness and reflection, to promote resilient leader/leadership adaptive patterns across various situations. Few empirical resilience studies have actively sought to gain from organizational disorder (Taleb, 2013), to expand a needed stress tolerance for leaders (Dannels and Masters, 2020), or in high performing contexts (Kegelaers et al., 2021; Fletcher and Sarkar, 2016) by combining exposure to naturally stressful circumstances with support and learning to develop needed resilience skills and behaviors. The key question is then identifying the threshold at which pressure becomes beneficial for the leaders during training processes, impacting chosen outcome indicators. When training adversity preparedness in the study, this threshold must during training be defined by the leaders’ own chosen cases and training opportunities.

With this study, we address the urgent need for rigorous empirical research in organizational resilience training, a field under-investigated and in early development (Scheuch et al., 2021). This study is the first of its kind, to conduct an ambitious randomized controlled trial (RCT) intervention specifically for leaders in non-military contexts, grounded in theory and incorporating cyclic short-term resilience sprints for an extended period. It adopts a longitudinal approach to ensure ample time for resilience development (Masten and Reed, 2002), responding to calls for a deeper understanding of outcome effects and change mechanisms (Robertson et al., 2015; Vanhove et al., 2016). Through questionnaires completed at multiple time points (T0, T1, T2), the study allows us to examine both longitudinal follow-up effects and development pathways, strengthening the understanding of leader resilience outcomes and resilience processes during training. Recognizing the interaction between individual and organizational context, the research will further qualitatively and quantitatively evaluate several categories of influential factors through mixed-methods process evaluation frameworks that may help explain intervention outcomes. Although voluminous qualitative interviews could strengthen this part of the study, we are careful not to burden the leaders and organizations too much during extremely challenging times, and we expect the overall mixed-method design to provide a comprehensive understanding of resilience building processes in pressured leader contexts.

To strengthen the study methods and meet reviewer recommendations (Robertson et al., 2015; Scheuch et al., 2021), the study incorporates the Resilience at Work (RAW) scale for enhanced contextual relevance. In combination with the widely used CD-risk resilience scale, it allows for capturing psychological leader resilience outcomes more precisely. While self-reported assessments have potential limitations, we will ensure the validity and efficiency of study methods to obtain reliable outcome data. Importantly, traditional resilience studies may use classical test theory and traditional psychometric methods, assuming equal measurement precision for all respondents. However, item response theory (IRT) accounts for the variability in measurement precision based on latent attribute values and aims to explain more variance in the data (Jabrayilov et al., 2016). The Rasch model establishes a consistent measurement scale across respondents by testing scale validity through person/item alignment (Stemler and Naples, 2021). In the study, evaluating “differential item functioning” ensures the uniformity and stability of outcome scales across leaders and item difficulty levels in the study. By adopting this model into the resilience study, we expect to enhance the precision of the employed scales, improve the quality monitoring, and ensure accurate processing of respondents’ performance (Boone, 2016).
