# Supplementary figures and images for: Psychological resilience training for leaders in extreme times: study protocol of a randomized controlled trial
Source: Front Psychol. 2025 Sep 22;16:1514954. doi: 10.3389/fpsyg.2025.1514954 (PMC12499496; doi:10.3389/fpsyg.2025.1514954)

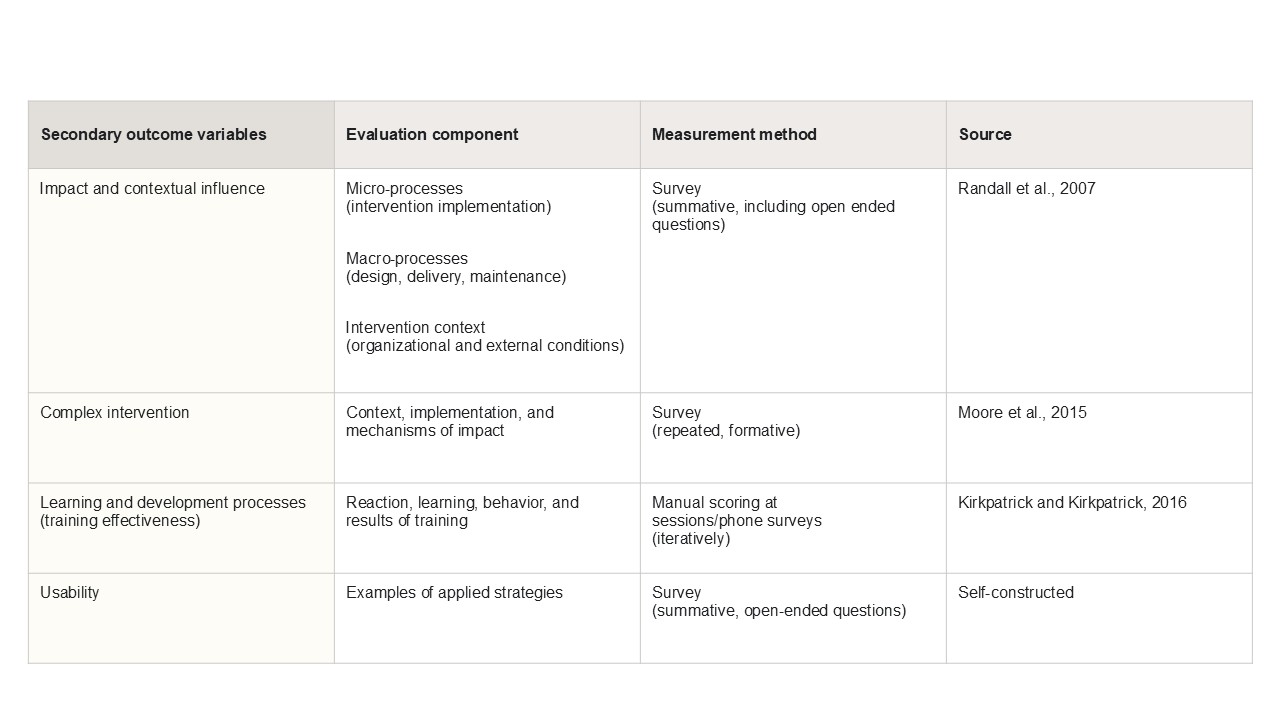

Supplement: Supplementary file 1 [file Supplementary_file_1.jpg]
