# Supplementary material for: Psychological resilience training for leaders in extreme times: study protocol of a randomized controlled trial
Source: Front Psychol. 2025 Sep 22;16:1514954. doi: 10.3389/fpsyg.2025.1514954 (PMC12499496; doi:10.3389/fpsyg.2025.1514954)
Supplement: Supplementary file 2 [file Supplementary_file_2.docx]

| **THE LEADER RESILIENCE TRAINING COMPONENTS** | | |
| --- | --- | --- |
| **Resilience component** | **Session** | **Description of Basic skills (B) and Advanced skills (A)** |
| Introduction | Kick-off | Introduction of program. Meeting the researchers, HR-trainers, and other participants |
| My Inner  (cognitive) | 1 | B. Mental awareness and change-skills  A. Mental her-and-now technique in difficulties |
|  | 2 | B. System 1 and 2. Logical thinking and mental biases  A. If-then logic and mental traps at work |
|  | 3 | B. Emotional reactions and mental rules  A. Casework: if-then thinking in leadership |
|  | 4 | B. Worries and mental realism  A. Change of perspective in catastrophic thinking |
|  | 5 | B. Technique to tackle an acutely stressful leader situation  A. Further training in techniques when under pressure |
|  | 6 | B. Calm down breathing and muscle relaxation  A. Stress reducing techniques during pressured periods |
| My strengths | 7 | B. Identification of leader strengths  A. Recognition of strengths in leadership |
|  | 8 | B. Balancing strengths in the leader context  A. Strength dynamics and environmental adaptions |
|  | 9 | B. A meaningful life - who I want to become  A. Leader identity and resilient leadership |
|  | 10 | B. Growth from the use of strengths in adversity  A. Identifying positive aspects of organizational problems |
|  | 11 | B. Supported by strengths in stressful leading situations  A. Readiness for strength use under pressure |
|  | 12 | B. Complexity in relational strength collisions  A. Complexity in relational strength synergies |
| My team | 13 | B. Healthy explanations. Case: a change project  A. Training explanation styles in leadership situations |
|  | 14 | B. Influence and control in the leadership role  A. Influence on leading the team |
|  | 15 | B. Believing I can reach my goals in teamwork  A. Beliefs and empowered actions from experiences |
|  | 16 | B. Identifying and regulating emotional states when leading  A. Balancing positivity and negativity for the team |
|  | 17 | B. Energy in relationships, networks and leadership  A. Emotional, mental and behavioral energy exchanges |
|  | 18 | B. Communicating and responding constructively as a team  A. Simulation of constructive vs destructive leader responses |
